# Research Progress on Low-Carbon Ironmaking Technologies for China’s Iron and Steel Industry Under the Carbon Peaking and Carbon Neutrality Goals

**DOI:** 10.3390/ma19153163

**Published:** 2026-07-23

**Authors:** Wenwen Liu, Renjie Zhang, Haokun Li, Yuanhong Qi

**Affiliations:** 1 State Key Laboratory for Advanced Iron and Steel Processes and Products, Central Iron and Steel Research Institute Co., Ltd., Beijing 100081, China; liuwenwen1239@163.com (W.L.);; 2 Technology Center, Taiyuan Iron and Steel (Group) Co., Ltd., Taiyuan 030003, China; lihk@baowugroup.com; 3 Faculty of Metallurgical and Energy Engineering, Kunming University of Science and Technology, Kunming 650093, China

**Keywords:** low-carbon ironmaking, blast furnace transformation, non-blast furnace ironmaking, hydrogen metallurgy, hydrogen-based shaft furnace, smelting reduction, hydrogen-based flash ironmaking, carbon neutrality

## Abstract

Under China’s “carbon peaking before 2030 and carbon neutrality before 2060” targets, the low-carbon transformation of the ironmaking stage—which contributes approximately 70% of the CO_2_ emitted per ton of steel in the dominant blast furnace–basic oxygen furnace (BF–BOF) route—is decisive for decarbonizing the steel industry. In contrast to earlier reviews that describe individual technologies in isolation, this review provides a structured, cross-technology synthesis: the main routes are classified into short-/medium-term low-carbon blast furnace technologies (hydrogen-rich carbon-recycling oxygen blast furnace, hydrogen-rich injection, biomass char injection and ultimate energy-efficiency measures) and medium-/long-term non-blast furnace technologies (hydrogen-based shaft furnace direct reduction, smelting reduction, rotary hearth furnace, fluidized-bed reduction and electric smelting reduction) and are then compared on a common set of quantitative indicators—CO_2_ mitigation, specific energy and hydrogen demand, technology readiness level (TRL) and relative cost. On this basis, emerging hydrogen-based flash ironmaking, developed from the interdisciplinary integration of “iron and steel–non-ferrous metallurgy–hydrogen metallurgy–plasma”, is critically assessed, with a clear separation between laboratory/pilot feasibility and industrial readiness. We conclude that low-carbon blast furnace technologies, owing to low retrofit cost and high maturity, will dominate near-term mitigation, whereas hydrogen-based shaft furnaces and, in the longer term, hydrogen-based flash ironmaking define the pathway toward near-zero-carbon ironmaking—conditional on the availability of green hydrogen and low-carbon electricity and on overcoming key engineering barriers.

## 1. Introduction

The “dual carbon” strategy of achieving carbon peaking before 2030 and carbon neutrality before 2060 is a major strategic deployment made by China based on the overall situation of global climate governance and the country’s long-term sustainable development, and it is also a solemn commitment made to the international community. The implementation of this strategy will not only promote the process of global climate governance and facilitate the construction of a community with a shared future for mankind but also force China’s industrial system to accelerate green transformation, break through the bottleneck of resource and environmental constraints, and consolidate the national energy security defense line.

As of 2026, the steel industry has become China’s second-largest source of carbon emissions after the power industry, with annual carbon emissions of approximately 1.82 billion tons of $CO_{2}$, accounting for 15–17% of the national total emissions, and it is the industry with the highest carbon emission intensity in the manufacturing sector [1,2,3,4,5,6]. China’s steel production pattern is dominated by the long-process route of “blast furnace–basic oxygen furnace”, accounting for approximately 90%. Among them, the blast furnace ironmaking stage, because it is highly dependent on coke as a reducing agent, heat source, and burden skeleton, contributes approximately 70% of the carbon emissions per ton of steel in the entire process [7,8,9,10,11,12]. Therefore, overcoming the technical problems of low-carbon blast furnace ironmaking and exploring new paths for zero-carbon ironmaking are the key requirements for the steel industry to achieve the “dual carbon” goals on schedule. These figures are indicative: recent authoritative estimates of the annual CO_2_ emissions of China’s steel industry vary between roughly 1.6 and 1.9 Gt depending on the accounting boundary, statistical year and data source, e.g., the China Iron and Steel Association, IEA and World Steel Association; the values should therefore be read as an order-of-magnitude range rather than a fixed number.

Based on this, researchers in the metallurgical field in China and internationally have carried out a large number of systematic studies and developed a diversified technology system covering two major directions: low-carbon blast furnace transformation and non-blast furnace ironmaking. This paper systematically reviews the principles, application progress, advantages, and disadvantages of current mainstream low-carbon ironmaking technologies and focuses on introducing the research achievements of hydrogen-based flash ironmaking technology proposed on the basis of interdisciplinary integration, so as to provide a reference for the development of deep decarbonization technologies in the steel industry.

### Scope and Review Methodology

This review follows a structured, though non-systematic, protocol. Peer-reviewed publications were retrieved from the Web of Science, Scopus and CNKI databases using combinations of the search terms “low-carbon ironmaking”, “hydrogen metallurgy”, “blast furnace”, “direct reduction”, “smelting reduction” and “flash ironmaking”, covering the period 2000–2026 with emphasis on the last five years. To capture engineering progress not yet reflected in the archival literature, Chinese and international industrial demonstration reports, corporate technical disclosures and national standards were additionally consulted. Sources were included when they reported the process principle, operating data, or techno-economic/environmental performance of an iron-ore reduction route relevant to decarbonization and were excluded when they addressed only economic or policy aspects without process content. Approximately 160 sources were finally retained. Throughout this paper and in Table 1, data drawn from demonstration projects or corporate releases are treated as indicative and are distinguished from peer-reviewed results, so that the reliability of each reported value can be judged by the reader.

Five categories of evidence are distinguished throughout the text and in Table 1, namely (i) peer-reviewed literature, (ii) industrial demonstration data, (iii) company press releases and corporate technical disclosures, (iv) theoretical or model-based estimates and (v) laboratory- and pilot-scale results; the category is indicated wherever a specific performance figure is quoted. The system boundary is likewise defined explicitly. Unless stated otherwise, all CO_2_ reduction values quoted in this review refer to direct process emissions of the ironmaking step (Scope 1, gate-to-gate, per ton of hot metal or DRI), relative to a conventional blast furnace baseline. Plant-level and full-process (BF–BOF or DRI–EAF) emissions and life-cycle emissions that include the upstream carbon intensity of hydrogen and electricity are reported separately and are identified as such. Accordingly, the descriptors “zero-carbon” and “near-zero-carbon” are used in this review only in the sense of direct process emissions and are valid at the plant and life-cycle levels only when green (electrolytic) hydrogen and low-carbon electricity are assumed; for hydrogen-, plasma- and electricity-intensive routes, this assumption dominates the reported carbon footprint and is stated wherever such values are given.

Compared with previously published reviews, which are largely descriptive and treat each technology in isolation, the distinctive contribution of the present work is threefold: (i) it places both low-carbon blast furnace and non-blast furnace routes within a single classification framework organized by deployment horizon; (ii) it compares the technologies on a common set of quantitative indicators—CO_2_ mitigation, specific energy and hydrogen demand, TRL and relative cost (Table 1)—rather than by qualitative judgement alone; and (iii) it positions the emerging hydrogen-based flash ironmaking route relative to the established options and separates its laboratory/pilot feasibility from its industrial readiness. Unless stated otherwise, the terms hot metal (liquid, carbon-saturated iron), direct reduced iron (DRI)/sponge iron (solid, porous, metallized product of gas-based reduction) and iron nuggets (coalesced, slag-separated metallic iron from rotary hearth processes) are used consistently in the sense defined here.

## 2. Research Progress of Low-Carbon Blast Furnace Ironmaking Technologies

Low-carbon blast furnace ironmaking reduces carbon emissions through process optimization, reducing-agent substitution, and energy recycling under the premise of maximizing the reuse of existing blast furnace infrastructure. It is currently the main technological choice for carbon reduction in the steel industry in the short and medium term.

### 2.1. Hydrogen-Rich Carbon Recycling Oxygen Blast Furnace Technology

**Figure 1:**
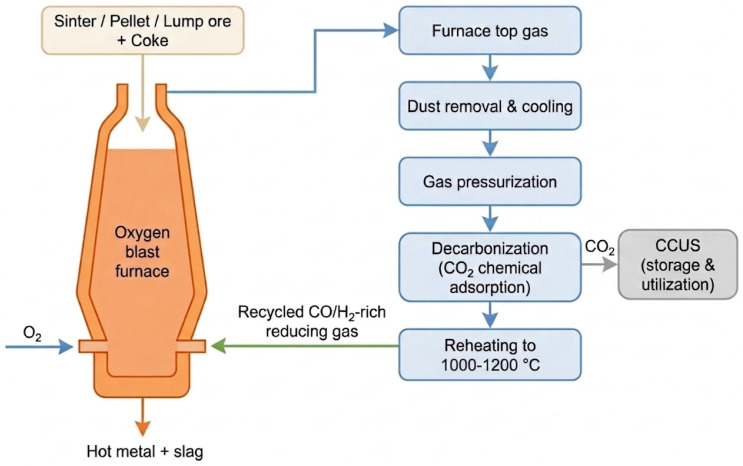
Process flow of HyCROF [13] (original schematic redrawn by the authors to avoid third-party copyright).

Hydrogen-rich carbon recycling oxygen blast furnace technology, namely HyCROF/OBF, is recognized as a core technology for the transition from traditional blast furnaces to zero-carbon ironmaking [13,14,15,16,17,18,19,20,21,22,23]. It is designed to achieve deep carbon reduction while retaining the main structure of the existing blast furnace. This technology replaces the hot blast used in traditional blast furnaces, which contains 79% nitrogen, with high-purity oxygen, eliminating the dilution effect of nitrogen on blast furnace gas and significantly increasing the calorific value of the gas. At the same time, based on the oxygen potential equilibrium principle of $CO/CO_{2}/C$, the composition of blast furnace gas is regulated, the proportion of $CO_{2}$ is reduced, and the concentrations of CO and $H_{2}$ are increased. After pressurization and purification, the produced blast furnace gas is separated from $CO_{2}$ through mature decarbonization technologies, such as chemical adsorption, and the separated $CO_{2}$ is delivered to the CCUS system for storage and utilization. The decarbonized $CO/H_{2}$-rich gas is heated to 1000–1200 °C and then blown back into the blast furnace through dedicated tuyeres to participate in the reduction cycle (Figure 1).

In October 2023, Xinjiang Bayi Iron & Steel of Baosteel Group completed the full-process commercial demonstration of this technology on a $2500\;m^{3}$ blast furnace [24,25,26]. The operation data show that, compared with a traditional blast furnace, its solid fuel consumption was reduced by approximately 30%, the blast furnace utilization coefficient increased by 40%, carbon emissions were reduced by more than 20%, and the annual $CO_{2}$ emission reduction of a single blast furnace reached 600,000–1,000,000 tons. The route offers a controllable retrofit cost and improved energy utilization efficiency. Its principal constraints are as follows: it cannot get rid of dependence on carbon-based energy and can only serve as a transitional technology; new systems, such as oxygen production, gas pressurization, decarbonization, and high-temperature heating, need to be added, which significantly complicates the process flow; the storage and transportation problems of high-temperature gas make large-scale promotion difficult; and the energy consumption of the newly added systems causes the overall comprehensive energy consumption to increase. From a techno-economic standpoint, the incremental capital cost of a HyCROF retrofit is reported to be far lower than that of a new hydrogen-based shaft furnace because the existing blast furnace shell is reused; however, the added oxygen plant, gas compression, CO_2_ capture and high-temperature reheating raise the operating cost and auxiliary energy demand relative to a conventional blast furnace. A rigorous comparison of capital investment, operating cost per ton of hot metal and CO_2_ mitigation cost is still lacking in the open literature and is summarized on an indicative basis in Table 1.

### 2.2. Hydrogen-Rich Injection Technology for Blast Furnaces

**Figure 2:**
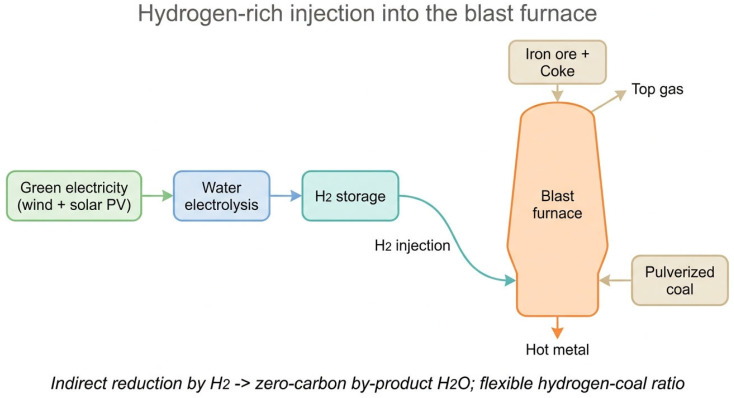
Hydrogen-enriched injection technology for blast furnace [32] (original schematic redrawn by the authors to avoid third-party copyright).

Hydrogen-rich injection technology for blast furnaces directly injects pure hydrogen into the existing blast furnace system to replace part of the carbon-based fuels and reducing agents, thereby reducing process carbon emissions [27,28,29,30,31,32,33,34,35,36,37,38,39,40,41,42,43]. It exploits the stronger reducing ability of hydrogen to strengthen the indirect reduction of iron oxides and generate the zero-carbon by-product water vapor. At the same time, it relies on the high calorific value of hydrogen to supplement the heat in the furnace, reduce the consumption of carbon-based fuels for heat compensation, and further improve the heat compensation efficiency in combination with oxygen-enriched combustion (Figure 2).

The industrial demonstration project of “green electricity water electrolysis hydrogen production-hydrogen storage-hydrogen-rich blast furnace smelting” has been operating continuously and stably. The operation results show that the pure hydrogen injection amount can reach $11,900\;m^{3}/h$, achieving a carbon reduction of 8–11% in the ironmaking stage [32]. The route requires minimal retrofitting, permits full reuse of existing blast furnace facilities, has a high technological maturity and operational safety, and allows the operator to adjust the hydrogen–coal ratio according to market and production requirements to control costs. However, this technology also cannot realize pure hydrogen ironmaking, and it requires precise control of the injection ratio based on a new hydrogen–coal balance model, which imposes high requirements on operational precision. A further constraint is the hydrogen supply chain itself: large-scale green-hydrogen production by water electrolysis is still energy-intensive and costly, and on-site storage, high-pressure transport and buffering to match the continuous, high-throughput demand of a blast furnace remain the main barriers to wider deployment. In addition, hydrogen injection alters the furnace thermal balance and lowers the raceway adiabatic flame temperature, so the injection ratio, hydrogen utilization efficiency and bosh gas volume must be controlled within narrow limits to preserve operational stability.

### 2.3. Biomass Char Injection Technology

**Figure 3:**
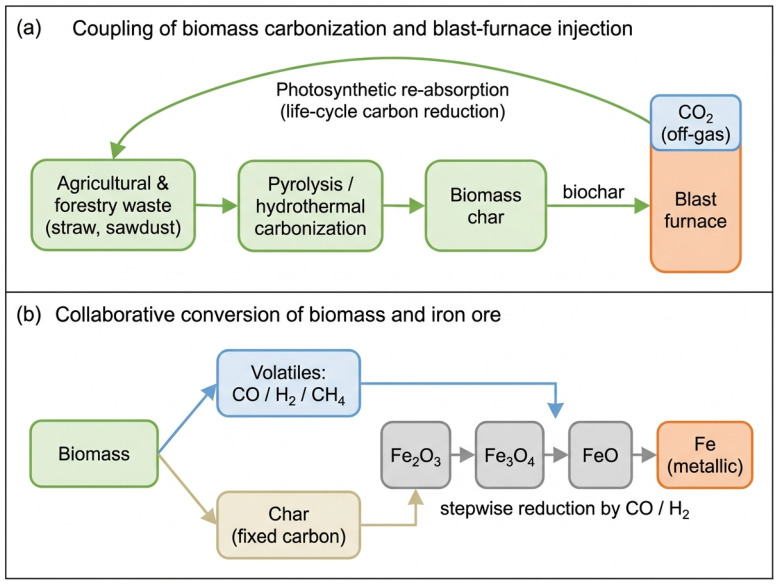
Biomass char injection technology [45] (original schematic redrawn by the authors after Ref. [45] to avoid third-party copyright). Note: (**a**) Coupling process of biomass gasification and iron ore reduction; (**b**) schematic diagram of transformation pathways in the collaborative conversion of biomass and iron ore.

Biomass char injection technology follows the concept of “treating waste with waste”. It converts agricultural and forestry wastes, such as straw and sawdust, into biomass char with a high carbon content through pyrolysis carbonization or hydrothermal carbonization processes, replacing fossil energy carbon used for blast furnace injection and achieving life-cycle carbon reduction [44,45,46,47,48,49,50,51,52,53,54,55,56,57] (Figure 3).

Brazil has long used charcoal to partially replace pulverized coal for blast furnace injection, reducing carbon emissions from blast furnace ironmaking by approximately 30% [50]. Biomass is a renewable resource, the comprehensive carbon consumption is low, the operating cost is controllable, and no modification of the existing blast furnace is required. At the same time, it realizes the resource utilization of agricultural and forestry wastes. The principal limitations are as follows: some biomass chars contain relatively high levels of alkali metal impurities, such as potassium and sodium, which easily cause corrosion and accretion of blast furnace linings; biomass raw materials are seasonal and scattered in distribution, and the collection, transportation, and storage systems are not yet complete; the calorific value of biomass char is lower than that of fossil coal, which will increase comprehensive energy consumption; and the raw materials need to undergo multiple pretreatment steps, such as drying and carbonization, resulting in a relatively long process flow. The life-cycle carbon neutrality of biomass char is, however, conditional. It depends on sustainable feedstock sourcing that avoids competition with food production and land-use change, on the logistics of collecting a seasonal and geographically dispersed resource, and on the ash chemistry of the char—particularly its alkali (K, Na) loading, which can accelerate lining corrosion and accretion. These factors must be included in any cradle-to-gate assessment before the route can be counted as genuinely carbon neutral.

### 2.4. Ultimate Energy Efficiency Technology for Traditional Blast Furnaces

Ultimate energy efficiency technology for traditional blast furnaces is a collection of technologies for tapping the carbon reduction potential of existing blast furnace systems. Its core is to achieve precise matching between material flow and energy flow in the blast furnace through process parameter optimization and intelligent control, thereby maximizing energy utilization efficiency [58,59,60,61,62,63,64,65,66,67,68,69,70,71,72,73,74]. Specific technical paths include constructing a “three-stage dust removal + forced ejector” gas recovery system and combining it with an intelligent control model to recover and utilize top-discharged blast furnace gas; using the pressure energy of blast furnace top gas for TRT turbine power generation [71], using physical condensation to remove moisture from the blast, where every $1\;g/m^{3}$ reduction in moisture can reduce the coke rate by 0.8–1.0 kg/t [63], based on the theory of the bosh gas volume index, optimizing the distribution of materials and gas flow in the furnace through an intelligent control system and extending the residence time of reducing agents; and adopting “induced erosion-type” hearth and bottom cooling technology to extend blast furnace service life and reduce the additional energy consumption caused by major repairs and furnace condition fluctuations.

The China Iron and Steel Association analyzed and statistically examined 58 leading enterprises with a production capacity of 440 million tons, showing that ultimate energy efficiency technology can reduce blast furnace energy consumption by 0.47% year-on-year [64]. This technology does not require modification of the blast furnace body and can achieve an average carbon emission reduction of 3–5%. Its main limitations are as follows: it involves complex fluid dynamics simulation and intelligent control algorithms, making management and control difficult; technologies such as high-proportion pellet smelting have relatively high requirements for iron ore quality; and the carbon reduction potential is limited, while the marginal investment cost of further carbon reduction will increase significantly. As a representative case, integrated ultimate-energy-efficiency measures (top-gas recovery, TRT power generation, blast dehumidification and intelligent burden control) applied at leading Chinese works are reported to lower the comprehensive energy consumption of ironmaking by the order of tens of kgce per ton of hot metal, corresponding to an indicative CO_2_ reduction of about 3–5% and an attractive payback owing to the low retrofit cost; precise, independently verified plant-level savings are, however, still scarce and are needed to substantiate the economic benefit.

## 3. Research Progress of Low-Carbon Non-Blast Furnace Ironmaking Technologies

Low-carbon non-blast furnace ironmaking technologies completely get rid of the dependence of traditional blast furnaces on coke. By changing the reactor form and reducing-agent type of the ironmaking reaction, they realize a higher degree of carbon emission reduction and constitute the core technological direction for the steel industry to achieve carbon neutrality in the medium and long term.

### 3.1. Hydrogen-Based Shaft Furnace Direct Reduction Technology

**Figure 4:**
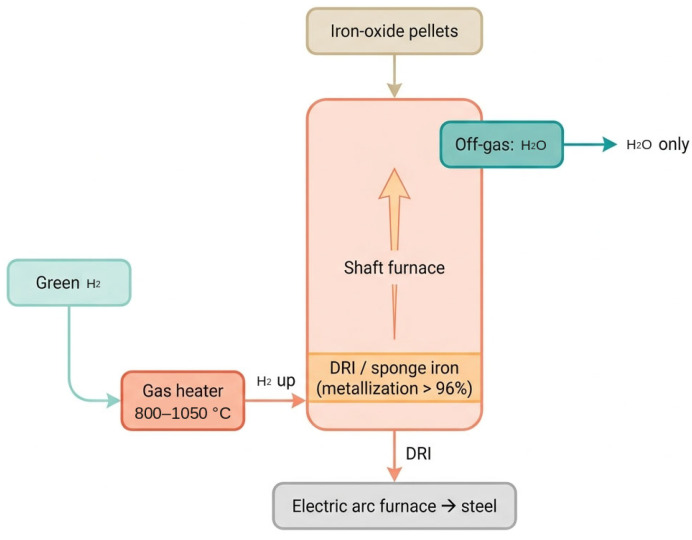
Hydrogen-based shaft furnace process flow diagram of Baowu Zhanjiang Iron and Steel [83] (original schematic redrawn by the authors to avoid third-party copyright).

Hydrogen-based shaft furnace direct reduction, namely $H_{2}$-DRI, is one of the core technologies for achieving carbon neutrality and even zero carbon in the ironmaking process. Its core is to completely replace the carbon-based reducing agents of traditional blast furnaces with pure hydrogen [75,76,77,78,79,80,81,82,83,84,85,86,87,88,89,90]. This technology uses a shaft furnace as the reaction vessel. High-grade iron ore is pelletized and sintered and then charged from the top of the furnace. Pure hydrogen or hydrogen-rich gas, such as coke oven gas or natural gas reforming gas, is heated to 800–1050 °C and blown in from the lower tuyeres. It contacts iron oxide pellets countercurrently to undergo reduction reactions, producing solid direct reduced iron, namely DRI, which is then transferred to an electric furnace to complete the steelmaking process (Figure 4).

China Baowu Zhanjiang Iron and Steel’s low-carbon steel project, with an annual output of 1.8 million tons based on “hydrogen-based shaft furnace + electric furnace”, was fully connected in December 2025. Operation data show that, compared with traditional blast furnace ironmaking, this technology reduces carbon emissions by more than 80% and shortens the process flow by approximately 40% [83]. In addition, HBIS Group Zhangxuan Technology’s 1.2 million-ton “hydrogen-based shaft furnace-near-zero-carbon electric arc furnace” short-process project, China Iron & Steel Research Institute’s pure hydrogen shaft furnace demonstration line in Linyi, Shandong, and the Swedish HYBRIT project have all achieved phased results [81,82,83,84]. The principal benefit of this route is its environmental cleanliness. When pure hydrogen is used, the flue gas contains only water vapor, and zero carbon emissions can be achieved in the ironmaking stage. The produced sponge iron has a metallization rate of more than 96% and a low impurity content, making it suitable for producing high-end steel products. Moreover, high-pollution and high-carbon-emission processes, such as sintering and coking, are eliminated, significantly shortening the process flow. However, this technology has strict requirements for raw materials. Because the smelting temperature in the furnace is low and there is no slagging stage, high-grade iron ore must be used, which limits its raw material adaptability. These performance figures are drawn from the operating enterprise’s public disclosures rather than from independent peer-reviewed measurement and should be read as demonstration-project data. It must further be stressed that the “>80% carbon reduction” refers to direct process emissions; from a life-cycle perspective, the overall environmental performance is governed by the carbon intensity of the hydrogen used, so the route approaches zero carbon only when green (electrolytic) hydrogen produced from low-carbon electricity is supplied.

### 3.2. Two-Step Smelting Reduction Technology

**Figure 5:**
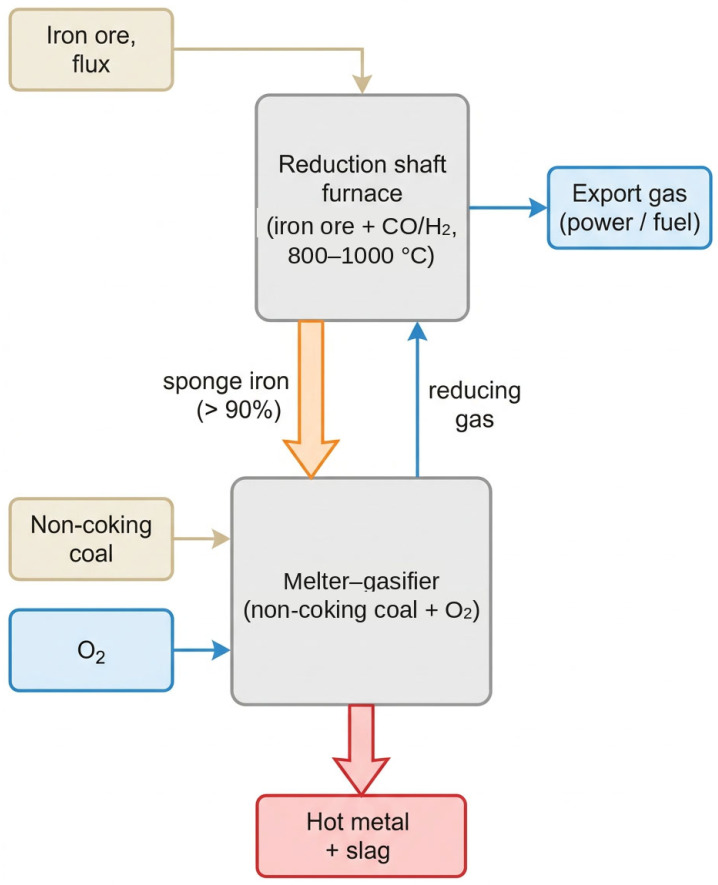
COREX process flow diagram [97] (original schematic redrawn by the authors to avoid third-party copyright).

Two-step smelting reduction technology divides the single-reactor ironmaking process of traditional blast furnaces into two stages: solid-state pre-reduction and smelting reduction. Typical processes include COREX, FINEX, and DIOS. In the solid-state pre-reduction stage, a reduction shaft furnace in COREX or a fluidized bed reactor in FINEX is used. Iron ore and high-temperature reducing gas, namely $CO+H_{2}$, contact countercurrently and are reduced at 800–1000 °C into sponge iron, with a metallization rate of more than 90%. The smelting reduction stage is carried out in a melter–gasifier. After sponge iron, non-coking coal, and flux are added, the sponge iron is melted, and slagging clarification is completed, producing molten hot metal and slag. Coal gasification provides reducing gas and heat for the pre-reduction stage [91,92,93,94,95,96,97,98,99,100,101,102,103] (Figure 5).

Baosteel introduced and improved COREX technology to develop the “Ouye furnace”. By optimizing gas pipelines and coal injection processes, it significantly improved the operation rate and reduced the cost per ton of iron, with the proportion of non-coking coal used exceeding 60% [97,98,99,100,101,102]. POSCO’s FINEX process in South Korea replaces the reduction shaft furnace with a fluidized bed [103], completely eliminating the sintering and pelletizing processes. The FINEX process eliminates coking and sintering, markedly reduces full-process carbon emissions, and can use non-coking coal and fine ore as raw materials. However, this technology is still dominated by coal-based reducing agents, and its carbon emissions are higher than those of other green non-blast furnace technologies. The COREX process has relatively high requirements for ore particle size and coal quality. The reduction shaft furnace is prone to sticking, refractory erosion is serious, and furnace life is short. The energy consumption per ton of iron is 2–4 GJ higher than that of a traditional blast furnace. In addition, gas production is large, and supporting processes such as power generation are needed to realize secondary energy utilization, so the overall energy utilization rate is relatively low [97,98,99,100,101,102]. On a systematic basis, FINEX generally shows a higher thermodynamic and carbon efficiency than COREX because it eliminates sintering and pelletizing and tolerates fine ore, whereas COREX retains a lump-burden shaft and higher specific energy consumption; both remain coal-based and therefore reduce CO_2_ only moderately relative to green non-blast furnace routes. Their industrial maturity is high (commercial units in operation), but their process economics are sensitive to coal price and to the value recovered from the large volume of export gas, as compared quantitatively in Table 1.

### 3.3. One-Step Smelting Reduction Technology

**Figure 6:**
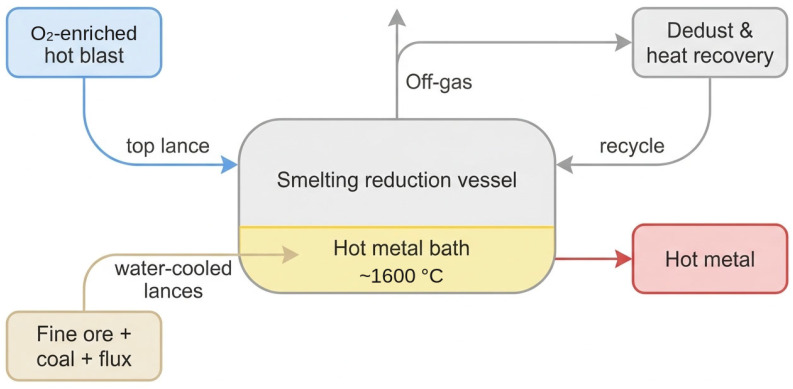
HIsmelt process flow [107] (original schematic redrawn by the authors to avoid third-party copyright).

One-step smelting reduction technology is represented by the HIsmelt process. It uses the heat of a high-temperature hot metal bath in a single reactor to directly melt and reduce powdered iron ore and non-coking coal instantaneously. During the process, iron ore fines with particle size less than 6 mm, non-coking coal powder, and flux are carried by nitrogen or oxygen and injected at high speed into the bottom of a hot metal bath at 1600 °C through water-cooled lances. Due to their large specific surface area and excellent kinetic conditions, the materials are rapidly reduced to metallic iron. During the upward movement of CO bubbles generated by coal powder gasification, they combust with the oxygen-enriched hot blast injected through the top lance and release heat, maintaining the high temperature of the molten bath. The produced high-temperature gas is subjected to dust removal and waste heat recovery. Part of it is recycled back to the furnace, and the rest is used for power generation or as fuel [104,105,106,107,108,109,110,111,112,113,114,115,116] (Figure 6).

Rio Tinto built a demonstration plant with an annual output of 800,000 tons in Kwinana, Australia [105,106]. The daily output of Shandong Molong’s HIsmelt project has exceeded 2000 tons, the continuous production days have exceeded 180 days, and the produced hot metal contains extremely low levels of harmful elements, such as phosphorus and sulfur. The route is very short and eliminates all raw material pretreatment processes, such as coking, sintering, and pelletizing. It has strong raw material adaptability and can directly use fine ore, low-grade ore, and solid wastes, such as iron-containing dust and sludge from steel plants. The product purity is high, and the investment cost is relatively low [107,108,109]. However, this technology still cannot get rid of carbon-based reducing agents, and its carbon reduction capacity is limited. Maintaining a high-temperature molten bath and treating high-temperature flue gas lead to relatively high comprehensive energy consumption. The reaction in the furnace is intense, gas-flow scouring and slag erosion are serious, and the furnace body has a short service life. The requirements for the injection system and the control accuracy of the oxygen–coal ratio are extremely high, and furnace condition fluctuations and splashing are likely to occur. In addition, the gas has a high dust content, and the flue gas treatment system is complex. Beyond these points, the long-term economics of HIsmelt are strongly affected by operational reliability: the intense in-bath reaction and slag foaming impose severe refractory degradation and frequent maintenance of the water-cooled lances and hearth, which shorten campaign life and raise the effective cost per ton of hot metal. Independent, long-duration operating data are still limited.

### 3.4. Rotary Hearth Furnace Technology

**Figure 7:**
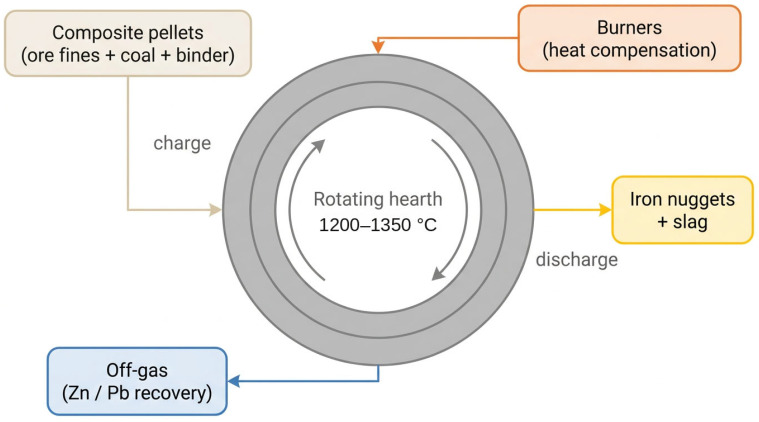
ITmk3 rotary hearth furnace process flow [118] (original schematic redrawn by the authors to avoid third-party copyright).

Rotary hearth furnace technology is represented by the Inmetco and ITmk3 processes, and its core equipment is a rotatable annular rotary hearth furnace [117,118,119,120,121,122,123,124,125,126,127]. During the process, iron ore fines or iron-containing dust and sludge are mixed with coal powder and binders and pressed into cold-bonded carbon-containing pellets, which are evenly distributed on the furnace hearth with a thickness of 100–200 mm. As the hearth rotates through a high-temperature zone of 1200–1350 °C, the coal powder inside the pellets reduces iron oxides to generate metallic iron, and the products are discharged from the outlet (Figure 7).

Shougang Jingtang’s 300,000-ton rotary hearth furnace is used to treat zinc-containing and iron-containing solid wastes, such as blast furnace dust and converter sludge, with a metallization rate of ≥75% and a dezincification rate of ≥90% [123]. Baowu Environmental Technology Shaogang’s project for treating 250,000 tons of zinc-containing dust and sludge annually achieved a dezincification rate of 88% [123]. Kobe Steel’s ITmk3 process in Japan can directly use ordinary iron ore or low-grade ore and produce iron nuggets with molten slag–iron separation [118]. The route has a very wide raw-material tolerance. It can treat low-grade complex ores, iron-containing solid wastes, and laterite nickel ore and can also comprehensively recover valuable metals, such as zinc and lead. The reaction speed is fast, the process is short, and the carbon reduction achieved is substantial. However, the single-furnace capacity of this technology is small, and it is difficult to scale up the furnace body, making it difficult to meet the production needs of large steel enterprises. A large number of burners are needed for heat compensation to rapidly heat the materials, so energy consumption is relatively high. In addition, the sulfur content in the product is difficult to control, and product quality stability is relatively poor. Environmentally, the process also raises secondary-waste concerns: the volatilized zinc- and lead-bearing dust collected downstream requires safe handling and further treatment, alkali and sulfur can accumulate in the recirculating streams, and the management of these by-products adds cost and must be accounted for when the route is presented as a clean solid-waste utilization technology.

### 3.5. Fluidized Bed Direct Reduction Technology

**Figure 8:**
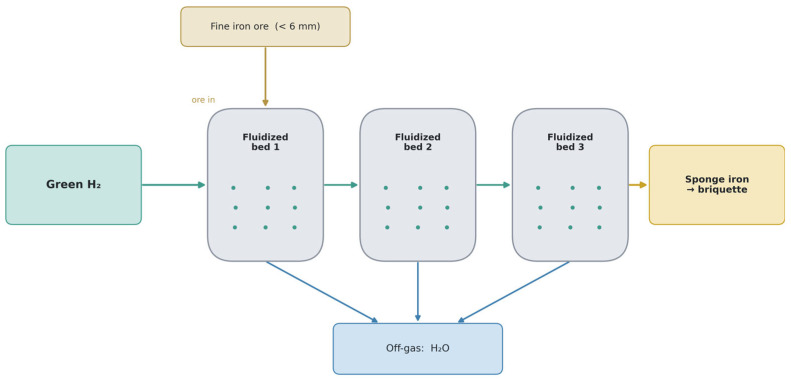
The process diagram of Ansteel fluidized bed direct reduction using green hydrogen [135] (original schematic redrawn by the authors to avoid third-party copyright).

Fluidized bed direct reduction technology is represented by Primetals Technologies’ HYFOR process. It uses reducing-gas back-blowing in a fluidized bed reactor to keep fine iron ore powder with a particle size of less than 6 mm in a fluidized state, thereby realizing efficient gas–solid contact reduction [128,129,130,131,132,133,134,135,136,137,138,139,140]. Reducing gas, such as natural gas or hydrogen, is introduced from the bottom of the reactor, and the flow velocity is controlled so that the ore powder is suspended. The extremely large specific surface area significantly improves heat and mass transfer efficiency. Iron oxides can be rapidly reduced into sponge iron, which is cooled, briquetted, and then sent to the steelmaking stage (Figure 8).

Million-ton fluidized bed direct reduced iron projects, namely the HIB and FIOR processes, have been built in Venezuela and Canada [135,136,137]. In 2024, Ansteel Group built a 10,000-ton green hydrogen fluidized bed demonstration line and achieved stable operation of the entire process, producing near-zero-carbon direct reduced iron, with a metallization rate of 95% [135,136,137]. Primetals Technologies’ HYFOR process has achieved 100% pure hydrogen reduction of iron ore powder [135,136,137]. The route provides a high gas–solid contact efficiency and a rapid reaction rate. It can directly use fine ore and eliminate the sintering and pelletizing processes. When pure hydrogen is used as the reducing agent, zero-carbon ironmaking can be achieved. However, this technology faces the core problem of “sticking-induced defluidization”; the sticky metallic iron generated on the particle surface during reduction easily causes particle sticking and leads to a “dead bed” in the fluidized bed. The high gas velocity leads to a high dust content in the tail gas. The smelting temperature is low, and the requirements for ore grade and harmful impurity content are strict, making it difficult to treat low-grade complex ores. In addition, to ensure the energy consumption level, multistage fluidized beds usually need to be connected in series, resulting in a relatively long process flow. Recent work has focused specifically on mitigating particle sticking, agglomeration and defluidization—for example, by surface coating or MgO/CaO modification of the ore, by controlling reduction temperature and gas velocity, and by pulsed or two-stage fluidization—which are the decisive barriers that still separate the route from stable, large-scale industrial operation.

### 3.6. Electric Smelting Reduction Technology

**Figure 9:**
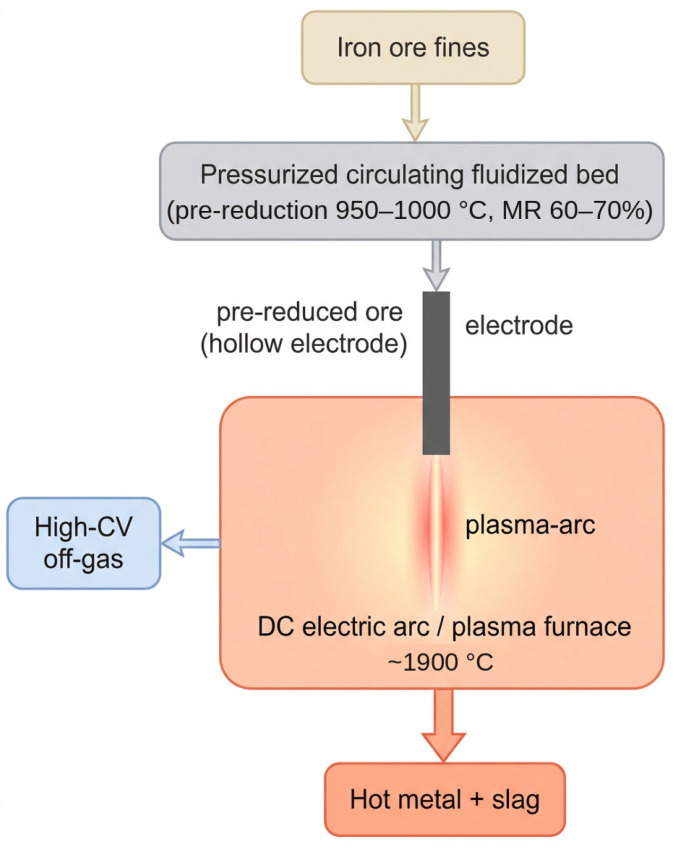
Flow sheet of hydrogen plasma reduction ironmaking [146] (original schematic redrawn by the authors to avoid third-party copyright).

Electric smelting reduction technology uses electric energy as the core heat source and uses the ultra-high-temperature characteristics of electric arcs or plasma, approximately 1900 °C, to break through the thermodynamic limitations of traditional carbothermic reduction and directly reduce iron ore into liquid hot metal. During the process, iron ore fines are first pre-reduced in a pressurized circulating fluidized bed. Reducing gas and fine coke particles generated by incomplete combustion of pulverized coal and preheated air are used to pre-reduce the ore at 950–1000 °C into pre-reduced ore, with a metallization rate of 60–70%. Then, the pre-reduced ore is added into the high-temperature reaction zone of a direct-current electric arc furnace through a hollow electrode. In the plasma environment of approximately 1900 °C, melting, reduction, carburization, and slagging are completed, producing molten hot metal and slag. Sweden has built a 25-ton reduction electric furnace test project, verifying the feasibility of this technology [141,142,143,144,145,146,147,148,149,150] (Figure 9).

The very high reaction temperature enables the route to treat complex iron ores that are difficult to process by conventional smelting methods. Coke is not required, and high-pollution links such as coking and sintering are eliminated. The flue gas has a high calorific value and a high CO concentration, making it easy to realize “iron-electricity” and “iron-chemical” co-production. The main drawbacks are as follows: the energy conversion efficiency is low, high electricity consumption leads to persistently high comprehensive energy consumption, the FeO content in the slag is approximately 12%, and the iron loss rate is relatively high. The sulfur content in the produced hot metal is approximately 0.3%, requiring external refining. High temperature and slag cause serious erosion of furnace lining refractory materials, and the furnace body has a short service life [146,147,148,149,150]. The industrial feasibility of this route is therefore tightly coupled to the power system: its high specific electricity demand makes both the achievable CO_2_ reduction and the operating cost strongly dependent on the availability of abundant renewable electricity and on regional electricity prices, so the route is attractive mainly in regions with low-cost low-carbon power.

## 4. Research Progress of Emerging Hydrogen-Based Flash Ironmaking Technology

### 4.1. Background and Process Principle of the Technology

In summary, countries around the world have developed a variety of low-carbon ironmaking technologies, but existing technologies all have shortcomings to varying degrees and are difficult to simultaneously meet the multiple requirements of carbon emission reduction, low energy consumption, high efficiency, short process route, and strong raw material adaptability. Therefore, they have not yet become the mainstream global ironmaking processes. Against this background, based on the interdisciplinary integration idea of “iron and steel-nonferrous metallurgy-hydrogen metallurgy-plasma”, hydrogen-based flash ironmaking technology has emerged. This technology combines the mature flash smelting technology of the nonferrous industry with hydrogen plasma technology and expands its application to the ironmaking field, providing a new technological path for realizing efficient zero-carbon ironmaking.

The core process of hydrogen-based flash ironmaking is as follows. Deeply dried iron ore concentrate is injected at high speed into the reduction tower of the flash iron furnace through a high-temperature plasma ore concentrate lance. In the tower space with high temperature and high reducing degree, the ore concentrate is in a highly dispersed state, forming “explosive” chemical kinetic conditions, and the multistage reduction reactions of iron oxides can be completed within several seconds. The generated metallic iron and slag directly fall into the smelting-separation bath at the lower part of the reduction tower and are discharged after slag–iron separation. The high-temperature flue gas generated in the process is mainly composed of water vapor. After waste heat recovery, dust removal, and dehydration, a small amount of unreacted hydrogen can be recovered and recycled. This technology can realize zero carbon emissions in the entire process and is especially suitable for treating low-grade complex ores that are difficult to smelt. At the same time, it completely eliminates traditional raw material preparation stages, such as sintering, pelletizing, and coking, and, in principle, offers a short process route, low energy consumption, high efficiency, precise control and favorable scale-up characteristics. It should be emphasized that these potential advantages have so far been demonstrated only at laboratory and pilot scale. The “zero-carbon” designation refers to direct process emissions and holds only when green hydrogen and low-carbon electricity are used; a full life-cycle assessment is therefore required before the route can be described as zero-carbon at plant level.

### 4.2. Current Research Status in China and Internationally

**Figure 10:**
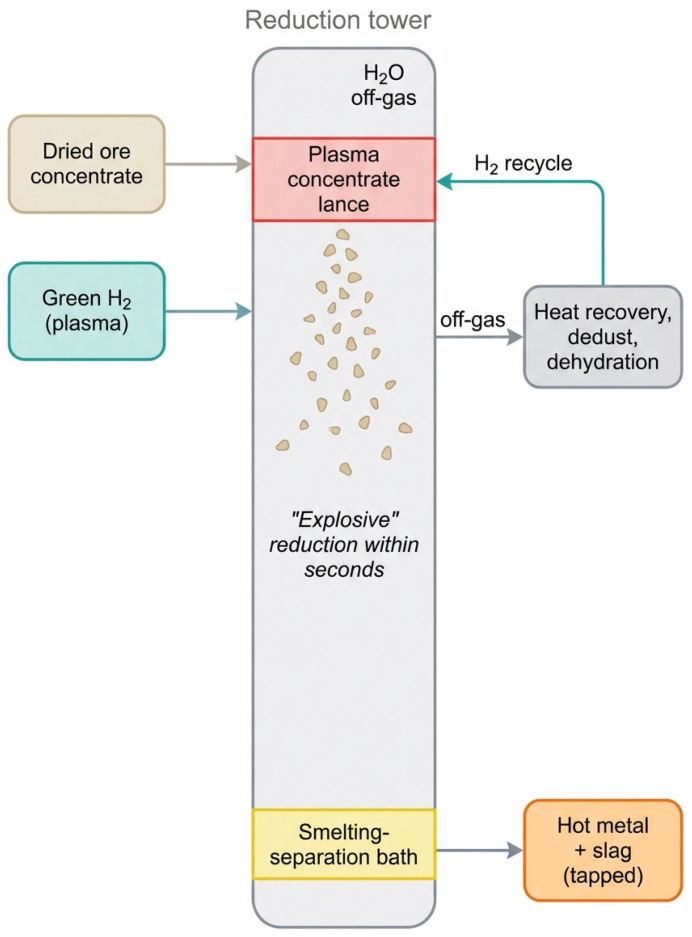
Flash ironmaking process flow sheet [151] (original schematic redrawn by the authors to avoid third-party copyright).

Hydrogen-based flash ironmaking technology has undergone more than ten years of basic research and experimental verification, and metallurgical scholars in China and internationally have constructed a relatively complete process theory system [151,152,153,154,155,156,157,158,159]. Professor H. Y. Sohn’s team at the University of Utah in the United States took the lead in conducting exploratory research on flash reduction of iron ore concentrate, proposed the concept of “suspension ironmaking”, and completed a large number of basic and pilot-scale tests. The team’s research shows that, when hydrogen is used as the reducing agent, under the conditions of 1175 °C and an ore concentrate residence time of several seconds, the reduction degree of iron ore concentrate can reach more than 90%. When natural gas is used as both reducing agent and fuel, the reactor temperature is increased to 1350 °C through partial combustion, and the reduction degree also exceeds 90% when the ore concentrate residence time is less than 3 s. Comparison of different feeding modes shows that the reduction degree of burner feeding is lower than that of side feeding. On this basis, the team independently developed a pilot test furnace of ∅0.08×H2.1 m. The pilot test results in 2019 showed that, under the conditions of an iron ore concentrate feeding rate of 2.5 kg/h, an average particle size of 45 μm, a natural gas flow rate of $25\;m^{3}/h$, an oxygen flow rate of $20\;m^{3}/h$, and a reduction temperature of 1250 °C, the iron ore concentrate could be reduced to sponge iron within several seconds, with a reduction degree of 94% (Figure 10).

Based on the research of Sohn’s team, the domestic project team innovatively optimized the structure of the flash iron furnace, realizing direct production of molten hot metal from flash reduction products and breaking through the limitation that traditional flash reduction can only produce sponge iron [160,161,162,163,164]. The team systematically studied the effects of parameters such as reducing agent flow rate and $H_{2}/CO$ ratio, reduction temperature, feeding rate, and ore concentrate characteristics on the reduction degree. It was found that, under the conditions of 1350–1500 °C and an ore concentrate residence time of less than 10 s, iron ore concentrate can be reduced to molten hot metal, with a reduction degree exceeding 90%. In terms of reaction kinetics, the team clarified that the reaction in the reduction tower of the flash iron furnace conforms to the interfacial chemical control model, established the corresponding chemical reaction kinetic equations, and quantified the effects of temperature, gas partial pressure, ore concentrate particle size, and heating rate on the reaction rate. At the same time, it clarified the matching mechanism between interfacial coalescence and reaction rate in the smelting-separation bath, constructed a prediction model for slag properties and a control method for carburization behavior, and developed a numerical simulation system for the flash ironmaking process. In terms of flow field simulation, the team established a multiphysics computational fluid dynamics model including turbulence, heat transfer, mass transfer, and chemical reactions, which can accurately predict the distribution of velocity field, temperature field, and material flow field in the furnace, and optimized the model parameters based on natural gas reduction conditions. In addition, the team also studied the effects of process and furnace structure parameters as well as refractory material selection on slag accretion on the furnace wall and completed the optimization of furnace body design. Based on the above research achievements, the team independently developed a pilot flash ironmaking test furnace of ∅1.2×H5.4 m, with a furnace tube size of ∅0.12×H2.7 m. The pilot test in 2018 verified the feasibility of this technology: at a reduction temperature of 1350–1500 °C, iron ore concentrate could be reduced to molten hot metal within 10 s, and the reduction degree was stabilized at more than 90%. Taken together, these results establish laboratory- and pilot-scale feasibility (broadly TRL 3–4) rather than industrial readiness. The reported outcomes originate mainly from a small number of research groups; a broader body of independent international studies, together with continuous long-duration operation at demonstration scale, is still required to confirm reproducibility. Critical scale-up and commercialization barriers remain, notably the service life of high-temperature plasma torches, stable slag–iron separation in the smelting-separation bath, and cascade utilization of system energy; these should be regarded as fundamental engineering challenges rather than minor refinements.

Beyond these two groups, an independent and geographically broader body of work now exists and is important for a balanced assessment. In Europe, the hydrogen plasma smelting reduction (HPSR) program of Montanuniversität Leoben and voestalpine (SuSteel) has demonstrated in-flight and in-bath reduction of iron oxides by thermal hydrogen plasma at laboratory and pilot scale and has quantified the strong effect of arc power, hydrogen fraction and slag chemistry on the reduction degree [146,147,148,149,150]. Comparable suspension- and flash-reduction studies have been reported for hydrogen–iron-oxide systems by groups in Germany (Max-Planck-Institut für Eisenforschung), Japan (ISIJ-affiliated laboratories, in the context of COURSE50) and Korea, as well as within the Swedish HYBRIT consortium, and confirm both the very fast intrinsic kinetics of fine-particle hydrogen reduction and the sensitivity of the process to particle size, heating rate and hydrogen partial pressure [151,152,153,154,155,156,157,158,159]. Taken together, the international evidence is consistent with the Chinese and US results reported above, but it also shows that no group has yet operated a continuous, integrated flash ironmaking unit for an extended campaign; the available data therefore support the process concept rather than its industrial readiness.

## 5. Multidimensional Comparative Analysis of Mainstream Low-Carbon Ironmaking Technologies

This section consolidates the technologies reviewed above into a single, cross-technology comparison. Table 1 summarizes each route against a common set of quantitative indicators—technology readiness level (TRL); CO_2_ mitigation relative to a conventional blast furnace; specific energy and hydrogen demand; industrial scale and status; and relative CAPEX/OPEX—together with the principal bottleneck and deployment horizon for each technology. Consistent with the system boundary defined in Section 1, the tabulated values refer to direct process (Scope 1) emissions unless otherwise indicated. On this basis the routes are grouped into short-/medium-term low-carbon blast furnace technologies and medium-/long-term non-blast furnace technologies.

## 6. Conclusions and Prospects

As a key field of China’s carbon emissions, the low-carbon transformation of the steel industry is a key link in achieving the “dual carbon” goals. At present, low-carbon blast furnace ironmaking technologies, with the advantages of low retrofit cost and high technological maturity, will become the main force for carbon reduction in the steel industry in the short and medium term. Non-blast furnace ironmaking technologies, such as hydrogen-based shaft furnaces, represent the development direction of medium- and long-term zero-carbon ironmaking. However, existing technologies all have their own limitations and are difficult to simultaneously meet the production requirements of the steel industry for large scale, low cost, and high efficiency.

Hydrogen-based flash ironmaking combines near-zero direct process emissions (conditional on a green-hydrogen and low-carbon-electricity supply), a wide raw-material tolerance, a short process route and a high reaction efficiency; it should therefore be regarded as a candidate long-term route rather than as a demonstrated industrial solution. At present, this technology has completed basic theoretical research and pilot verification. In the future, it is necessary to further carry out industrial-scale demonstration project construction; focus on solving key engineering problems, such as the service life of high-temperature plasma torches, efficient slag–iron separation in the smelting-separation bath, and cascade utilization of system energy; and promote the industrial application of this technology, so as to support the deep decarbonization of the steel industry. In stating these prospects, we distinguish explicitly between the demonstrated pilot-scale feasibility of the route and its industrial readiness, which has not yet been achieved.

On the basis of the comparative analysis (Table 1), the following ranked priorities are proposed. (1) Before 2030, the most realistic large-scale mitigation will come from low-carbon blast furnace technologies—ultimate energy efficiency together with hydrogen-rich and biomass char injection—owing to their low retrofit cost and high maturity; these are best suited to the large existing fleet of brownfield BF–BOF plants. (2) HyCROF-type oxygen blast furnaces coupled with CCUS are the preferred deeper-reduction retrofit where CO_2_ transport and storage infrastructure is available. (3) In the medium term, hydrogen-based shaft furnace direct reduction is the most mature near-zero-carbon route for new-build capacity, but it depends on high-grade ore/pellets and on a reliable supply of green hydrogen and low-carbon electricity. (4) Fluidized-bed and electric smelting reduction can extend green ironmaking to fine and low-grade ores once sticking/defluidization and electricity-cost constraints are overcome. (5) Hydrogen-based flash ironmaking is the most promising long-term route for China’s ore base but is presently at pilot scale; the first R&D priorities are plasma-torch service life, slag–iron separation and system-energy cascade utilization, followed by construction of an integrated demonstration plant. Across all routes, the availability of affordable green hydrogen and low-carbon electricity, together with transparent full-process (life-cycle) carbon accounting, will ultimately determine the pace of decarbonization.

## Figures and Tables

**Table 1:** Multidimensional comparative analysis of mainstream low-carbon ironmaking technologies. The table has been rebuilt to include quantitative indicators (TRL, CO_2_ reduction, specific energy and hydrogen demand, and relative CAPEX/OPEX).

Technology (Route)	TRL	CO_2_ Reduction vs. BF	Specific Energy Consumption	Reductant/H_2_ Demand	Industrial Scale/Status	CAPEX (Rel.)	OPEX (Rel.)	Key Bottleneck and Deployment Horizon
Hydrogen-rich carbon-recycling oxygen BF (HyCROF)	7	~20–30% (higher with CCUS)	~13–14 GJ/t HM + auxiliaries	Coke + recycled CO/H_2_-rich gas	Commercial demo, 2500 m^3^ BF (Bayi Steel)	Medium	Medium–high	CCUS and O_2_/reheating cost; short–medium term
Hydrogen-rich injection into BF	7–8	~8–11% (up to ~20%)	≈BF baseline	~10–30 kg H_2_/t (partial)	Stable industrial demonstration	Low	Medium	Green-H_2_ supply; flame-temperature/thermal balance; short term
Biomass char injection	6–7	~30% (up to ~40%)	≈BF, slightly higher	Biochar (fossil-C substitute)	Commercial in Brazil; pilot in China	Low	Medium	Feedstock/land-use, alkali, LCA; short term
Ultimate energy efficiency of BF	9	~3–5%	Slightly below BF baseline	Coke (unchanged)	Wide commercial application	Low	Low	Limited residual potential; available now
Hydrogen-based shaft furnace (H_2_-DRI)	7–8	>80% (up to ~95% green H_2_)	~10–12 GJ/t DRI + EAF power	~50–65 kg H_2_/t (~550–650 Nm^3^/t)	1.8 Mt/y project (Baowu Zhanjiang)	High	High	High-grade ore + green H_2_; medium term
Two-step smelting reduction (COREX/FINEX)	8–9	~20–30%	~2–4 GJ/t above BF	Non-coking coal (+CO/H_2_)	Commercial (Baosteel Ouye; POSCO FINEX)	High	Medium	Coal-based, energy penalty; available now
One-step smelting reduction (HIsmelt)	6–7	~15–25%	High (bath + off-gas)	Non-coking coal	Stable operation (Shandong Molong)	Medium–high	Medium–high	Refractory/campaign life; medium term
Rotary hearth furnace (ITmk3/Inmetco)	8	~20–30%	High (burner-intensive)	Coal in composite pellet	Commercial for Zn-bearing wastes	Medium	High	Small scale, secondary waste; niche now
Fluidized-bed direct reduction (HYFOR)	5–6	>70% (up to ~95% pure H_2_)	Moderate	~550–650 Nm^3^ H_2_/t (pure H_2_)	10 kt/y green-H_2_ demo (Ansteel); HYFOR pilot	Medium–high	High	Sticking/defluidization; medium term
Electric smelting reduction (plasma/EAF)	4–5	>50% (green electricity)	~3.5–4 MWh/t electricity	Electricity + limited C	25-t pilot (Sweden)	Very high	Very high	Electricity cost and TRL; long term
Hydrogen-based flash ironmaking	3–4	~90–100% (green H_2_ + power)	Low (reported, pilot)	~54–65 kg H_2_/t (stoichiometric+)	Pilot furnace (China/USA), reduction ≥90%	Medium–high	Medium–high	Torch life, slag–iron separation, scale-up; long term

Notes: TRL, technology readiness level; CAPEX/OPEX, capital/operating expenditure (qualitative, relative to a conventional blast furnace); BF, blast furnace; HM, hot metal; DRI, direct reduced iron; EAF, electric arc furnace; CCUS, carbon capture, utilization and storage. Quantitative values are indicative order-of-magnitude ranges compiled from the cited literature and demonstration-project reports; they depend strongly on plant configuration, ore grade and the carbon intensity of the hydrogen and electricity supply and should be verified against the specific cited sources. “Zero/near-zero carbon” values assume green hydrogen and low-carbon electricity.

## Data Availability

No new data were created or analyzed in this study. Data sharing is not applicable to this article.
